# Plant Foods and Underutilized Fruits as Source of Functional Food Ingredients: Chemical Composition, Quality Traits, and Biological Properties

**DOI:** 10.3390/foods9101474

**Published:** 2020-10-15

**Authors:** Dario Donno, Federica Turrini

**Affiliations:** 1 Dipartimento di Scienze Agrarie, Forestali e Alimentari, Università degli Studi di Torino, 10095 Grugliasco (TO), Italy; 2 Dipartimento di Farmacia, Università degli Studi di Genova, 16132 Genova, Italy; turrini@difar.unige.it

**Keywords:** natural plant foods, healthy properties, phytochemicals, agrobiodiversity, human nutrition, analytical strategies, bioactivity, unconventional fruits, in vitro test, natural antioxidants

## Abstract

Changes in lifestyle and demographics, rising consumer incomes, and shifting preferences due to advanced knowledge about the relationships between food and health contribute to generate new needs in the food supply. Today, the role of food is not only intended as hunger satisfaction and nutrient supply but also as an opportunity to prevent nutrition-related diseases and improve physical and mental well-being. For this reason, there is a growing interest in the novel or less well-known plant foods that offer an opportunity for health maintenance. Recently, interest in plant foods and underutilized fruits is continuously growing, and agrobiodiversity exploitation offers effective and extraordinary potentialities. Plant foods could be an important source of health-promoting compounds and functional food ingredients with beneficial properties: the description of the quality and physicochemical traits, the identification and quantification of bioactive compounds, and the evaluation of their biological activities are important to assess plant food efficacy as functional foods or source of food supplement ingredients.

It is known that specific foods confer additional health benefits to human beings as treatment and/or prevention of several diseases [1]. For this reason, people have achieved a better life quality by eating fruits, vegetables, and other foods derived from plants, and using nutraceuticals, dietary supplements, or nutritional phytotherapy following official medicine. Moreover, regional regulatory bodies stimulate high global development and research to identify new phytochemicals to be used in innovative nutraceuticals and functional foods [2]. “Nutraceuticals” may be defined as a food or part of a food that provides health-promoting benefits, and are used as adjuvants in several diseases [3]. Nutraceuticals are the fastest growing sector of the food industry with a market estimated between USD 6 billion and USD 60 billion [4] (5% growing per annum). However, there is still much confusion about nutrition in the population. In Europe and the USA, approximately 70% of people would buy specific foods to reduce the risk of diseases, but they are unable to follow dietary guidelines [5].

Nutraceuticals are detailed as food products purified, produced, or extracted from an animal or plant source (e.g., antioxidants from fish oils, blueberries, elk velvet), or produced from pressed, powdered, or dried plant material and demonstrated to present a health-promoting benefit or to protect against several chronic diseases [3,4]. Dietary supplements have more specific health roles (e.g., minerals, herbs or other botanicals, amino acids, vitamins, and other dietary ingredients) to supplement the diet by increasing the total intake of these substances [6], but they are not intended to treat disease [7]. Functional food is different from nutraceuticals and may be defined as food products used in the common diet to add beneficial health effects to the traditional nutritional ones [8]. It is important to define the key relations between the proposed concepts (nutrition, health, and technology) with the main actors involved in the processes and studies for the functional food development, namely: the nutritionist, the specialist, and the food technologist. The combination of their different skills is essential for the innovative development of these productions. These products, aimed at the maintenance of well-being, should present the highest quality standards if compared to the relative conventional products [9].

In the plant biochemistry, secondary metabolites (e.g., vitamins, polyphenols, terpenoids, organic acids), produced in the plants, are not directly involved in the normal organism growth and development, but they act as defense compounds against predators, diseases, parasites, ultraviolet radiation, and oxidants to facilitate the reproductive processes [10]. These same molecules show specific health-promoting benefits and effects in men and animals; clinical, epidemiological, in vitro, and in vivo studies have demonstrated that a diet rich in plant foods may reduce the risk of some degenerative diseases. These secondary plant metabolites with low molecular weight present excellent antioxidant and anti-inflammatory properties even if their action mechanisms vary greatly depending on the chemical structure and environment [11].

Today, nutraceuticals on the market consist of both traditional foods (e.g., vegetables, fruits, meat products, fish, grains, chocolate, and tea) and non-traditional foods (e.g., added ingredients and/or nutrients, or products derived from agricultural breeding). [4]. In recent years, wild plants (neglected and underutilized plants) have become essential for the food industry, thanks to their use as an alternative for synthetic nutraceuticals and chemicals [12], but their socio-cultural, economic, and nutritional potentials are still not fully exploited [13,14]; data on the antioxidant and health-promoting properties of several natural resources, as plants not used in medicine and nutrition, still lack. Studies on these plant materials are of interest to find innovative sources for natural nutraceuticals, antioxidants, and functional foods [15]. Recent studies in the field of natural biomolecules are increasing knowledge on naturally health-promoting substances available in food, mainly in fruit. Their use in food products may increase added-value and quality; new methodologies for extraction/purification and identification/quantification of bioactive compounds using ecofriendly analytical strategies need to be developed to improve the production yields [16,17].

This Special Issue provides readers with a good overview of the status and exciting developments in this field. It includes papers focused on modern analytical instrumentation and new methods and biological tests applied to the evaluation of underutilized plants and phytochemical characterization of innovative natural sources of bioactive compounds and relative health-promoting properties.

Fahad Alderees et al. investigated the bioactive composition of different extracts of *Tasmannia lanceolata*, *Backhousia citriodora*, and *Syzygium anisatum* by ultra-high-performance liquid chromatography and the mechanism of action against food spoilage yeasts together to their antioxidant and antimicrobial activities. The extracts showed broad-spectrum antifungal activity against weak-acid resistant yeasts in comparison to the standard antifungal agents. Polygodial, citral, and anethole were the main bioactive molecules identified in *Tasmannia lanceolata*, *Backhousia citriodora*, and *Syzygium anisatum*, respectively. The ethanol and methanol extracts showed the highest polyphenolic content and antioxidant properties, while the hexane extracts contained the highest amount of total bioactive compounds and demonstrated the strongest antimicrobial activities.

Charoonsri Chusak et al. studied the influence of the extracts from *Clitoria ternatea* L. flowers on the inhibition of pancreatic α-amylase and the starch in vitro enzymatic digestibility and predicted the glycemic index of different flours, such as potato, rice, wheat, glutinous rice, corn, and cassava flours. Moreover, the application in a bakery product, prepared from the studied flours and extracts, was also determined. The results showed that the extracts inhibited the pancreatic α-amylase activity together to a significant reduction in the glucose release, hydrolysis index (HI), and predicted glycemic index (pGI) of the considered flours.

Selina A. Fyfe et al. investigated the health-promoting properties (antioxidant capacity and antimicrobial activity), functionality, and phytochemical composition of the Australian Native Green Plum, *Buchanania obovata* Engl., evaluating its potential as a functional ingredient in innovative food products. The seed and flesh contained several polyphenols, such as ellagic acid, p-coumaric acid, gallic acid, trans-ferulic acid, quercetin, and kaempferol, that may be responsible for the biological activities. In particular, the seed, eaten as bush food, presented a delphinidin-based anthocyanin.

Saleha Akter et al. showed the chemical and nutritional composition of the kernels of a native Australian fruit, *Terminalia ferdinandiana* (vernacular name: Kakadu Plum), as a novel nutritional source. The food industry processes the *T. ferdinandiana* fruits into puree generating seeds as a by-product that is generally discarded. This study was aimed to process the Kakadu Plum seeds separating the kernel and determining its nutritional composition. *T. ferdinandiana* kernels presented the potential to be used as a new protein source for dietary purposes and non-conventional supply of palmitic, oleic, and linoleic acids.

Yang Cao et al. presented a review on the phytochemical composition, biological properties, and nutrigenomic implications of yacon as a potential source of prebiotic, evaluating the current evidence and future directions. Yacon is an underutilized plant consumed as a traditional root-based fruit in South America and it mainly contains fructooligosaccharides (FOS) and inulin. Therefore, it has bifidogenic benefits for gut health because FOS are not easily broken down by digestive enzymes. Scientific studies on the bioactive molecules and nutrigenomic properties of the extracts and isolated compounds from yacon may help in further research to investigate yacon-based nutritional products.

Underutilized and alternative fruits represent an excellent opportunity for local growers to gain access to special or niche markets where consumers appreciate exotic traits and the presence of nutrients able to prevent degenerative diseases. The creation of specific horticultural models for fruit production may be an important opportunity to obtain a high-standardized raw material and produce high-quality fresh or derived-products. Additionally, the phytochemicals extracted from these fruits could have an excellent application in the food industry for increasing the shelf life and stability of the commercial products. Several strategies should be applied to study: (i) the toxicological traits of bioactive extracts, (ii) the metabolism of bioactive compounds (including their bioaccessibility and bioavailability), and (iii) the sensory and nutritional traits of the food products added with biologically active molecules from underutilized fruits [18]. The economic evaluation of the extraction and marketing processes should be also contemplated because these products should be environmentally safe, non-environmentally impacting, and economical [19,20].

Studies on the isolation and characterization of bioactive compounds using complementary analytical methods and on their influence on biological status in animal/human models are needed for the evaluation of their potential benefits. Finally, it is important to further confirm the lack of toxicity of these sources together to their natural bioavailability [21].
